# Collagen Fibrils in Cultured and Wild Sea Bream (*Sparus aurata*) Liver. An Electron Microscopy and Image Analysis Study

**DOI:** 10.1100/tsw.2011.96

**Published:** 2011-04-19

**Authors:** Panagiotis Berillis, Eleni Mente, Ioannis Nengas

**Affiliations:** ^1^ Department of Ichthyology and Aquatic Environment, School of Agricultural Sciences, University of Thessaly, Nea Ionia, Magnisia, Greece; ^2^ School of Biological Sciences (Zoology), University of Aberdeen, Aberdeen, UK; ^3^ Hellenic Centre for Marine Research, Institute of Aquaculture, Agios Kosmas, Athens, Greece

**Keywords:** electron microscopy, image analysis, sea bream, collagen fibrils

## Abstract

This study aims to measure liver collagen fibril diameter in cultured and wild sea breams (*Sparus aurata*). Cultured sea breams were fed three isonitrogenous diets. The organically produced feed contained sustainable certified fish meal (45%), fish oil (14%), and organic certified wheat; the laboratory feed contained fish meal (45%), fish oil (14%), wheat meal, and soya meal; and the commercial feed included fish meal (46%), fish oil (17%), soya meal, wheat meal, and corn gluten meal. The organic diet had higher amounts of vitamins A, C, and E; specific amino acids; and minerals that enhanced the biosynthesis of collagen. This study shows that fish fed the organic feed had significantly bigger collagen fibril diameters than the fish fed the conventional feed. Furthermore, the organically fed fish had similarly sized collagen fibril diameters as wild fish. More research is needed to understand the long-term effects and the mechanism and function of fish collagen peptide intake on lipid absorption and metabolism; and to identify dietary regimes that are able to improve whole body lipid profiles and suppress the transient increase of plasma triglycerides.

## INTRODUCTION

Collagen is a group of naturally occurring proteins. It is abundant in most invertebrates and vertebrates[1,2]. It is the main protein of connective tissue and represents about one-fourth of the total protein content in many multicellular animals[3]. The collagen molecule is formed by three polypeptide strands, the alpha chains, each possessing the conformation of a left-handed helix. These three left-handed helices are twisted together into a right-handed coiled coil, a triple helix. There is some covalent cross-linking within the triple helices and a variable amount of covalent cross-linking between collagen molecule helices forming well-organized aggregates, such as fibrils[4]. Collagen fibers are bundles of fibrils. These fibrils are semi-crystalline aggregates of collagen molecules. Collagen exists in many places throughout the body (skin, bones, liver, and muscles). So far, 29 types of collagen have been identified and described. However, over 90% of the collagen in the body is of type I, II, III, and IV. Many fish have a specific type I collagen that is different from the type I collagen seen in higher vertebrates, such as humans, cattle, pigs, and chickens, and which includes a characteristic subunit that is not seen in higher vertebrates[5].

Several papers on invertebrate collagen emphasize its morphological and functional characteristics[6]. Collagen is one of the long, fibrous structural proteins whose functions are different from those of globular proteins such as enzymes. Tough bundles of collagen (collagen fibers) are a major component of the extracellular matrix that supports most tissues and gives cells structure from the outside. Collagen is also found inside some cells. It has great tensile strength[7,8]. Tzaphlidou and colleagues have studied the D-period and the fibril diameter in many tissues in order to determinate how collagen affiliates with tissue disorders or how it affects their mechanical properties[9,10,11,12].

The chemical and physical properties of collagen proteins in fishes are different in tissues such as skin, swim bladder, and the myocommata in muscle[13]. In general, collagen fibrils form a delicate network structure with varying complexity in the different connective tissues in a pattern similar to that found in mammals. The collagen in fish is much more thermolabile and contains fewer, but more labile cross-links compared to the collagen from warm-blooded vertebrates. In general, the hydroxyprolin content is lower in fish than in mammals, although a total variation between 4.7 and 10% of the collagen is observed[14]. Different fish species contain varying amounts of collagen in their body tissues. This has led to a theory that the distribution of collagen may reflect the swimming behavior of the species[15].

Over the past decade, sea bream (*Sparus aurata*) production in the Mediterranean Sea has increased dramatically, reaching more than 110,000 tons[16]. However, sustainable aquaculture development should consider the relative stability in the supply of fish meal and fish oil, and further research is needed to reduce the pressure on marine resources. Improvements in fish feed development and technology are necessary for the further increase in marine finfish production. Kaushik et al.[17] suggest that an almost total replacement of fish meal by vegetable ingredients in the sea bass diet does not affect fish growth or feed utilization. Gomez-Requeni et al.[18] suggest that a 50 and 75% replacement of fish meal by plant proteins sources in the juvenile sea bream diet decreased growth performance, and a total substitution of fish meal reduced growth performance by 30%. Sea bream fed a fish meal diet have a lower lipid content in their livers compared with sea bream fed a plant protein source diet (corn gluten meal, peas, rapeseed meal, and wheat gluten meal)[19]. However, the feed efficiency and protein efficiency ratio is significantly higher in sea bream fed the plant protein source diet and feed intake is higher in sea bream fed the fish meal diet. Cultured sea bream have a higher lipid content in their muscle and liver tissues than wild fish[20,21]. The diet composition and the feeding regime affect the chemical composition of fish tissues[22].

The liver is the largest internal organ, making up about 1% of total body mass in Atlantic salmon *Salmo salar*. It is an organ most often studied in fish. The livers of *S. aurata* have large contents of lipids and a well-developed biliary tract. Biliary tract structures were associated with dietary habits. Ultrastructually, the hepatocyes of *S. aurata* have a spherical nucleus with euchromosome and heterochromosome, prominent nucleolus, and nuclear membranes and pores[23]. The fish liver plays a central role in fish physiology, both in anabolic (proteins, lipids, and carbohydrates) and catabolic (nitrogen, glycogenolysis, etc.) metabolism. It is important for the metabolism of nutrients absorbed in the digestive tract, but also for the metabolism and detoxification of many toxicants accompanying the foodstuff[24]. The fish liver can store lipids, carbohydrates, vitamin A, and iron[25]. The depot fat in the fish’s body is formed from a surplus of calories in the diet, although changes in physiological activity affect the fat content of fish (gonad maturation). The fish liver is an interesting organ for the study of interactions between dietary manipulation and hepatic structure, morphology, and their functions. These hepatic alterations reflect on fish health and such responses allow the fish liver to be considered as a good indicator of fish health status. Many researchers have found that some trace elements and vitamins play an important role in collagen synthesis, metabolism, and in fibril diameter[26,27,28,29,30, 31,32,33,34]. Thus, the diet can affect fish collagen fibril diameter, at the ultrastructural level, in the liver. The liver is a target organ in the study of diet-induced effects as it is the main metabolic organ and plays an important role in fish immune responses.

Seafood products are the only significant source in the human diet of polyunsaturated fatty acids (n-3 fatty acids). Their associated health benefits are documented in the literature[35]. The nutritional characterization of aquaculture products represents an important step towards a quality certification of fish and seafood products for human consumption. It is also suggested that food-derived collagen peptides act as a biological trigger for collagen synthesis in humans[36].

The aim of this study is to evaluate if a fish meal–exclusive protein source diet, formulated with certified organic wheat meal and with lower fish oil content, affects the liver collagen fibers of cultured sea bream in a differential fashion compared to a fish meal, soybean/corn gluten/wheat meal diet. Furthermore, liver collagen fibers in wild gilthead sea bream will be examined and compared with the above cultured sea bream. To the knowledge of the authors, this is the first demonstration that collagen fibril diameters measured in the liver are affected by the nutritional status of fish.

## MATERIALS AND METHODS

### Experimental Animals and Diets

Sea breams (*S. aurata*) were obtained from a commercial fish farm in Greece and reared at the laboratory of Fish Nutrition and Pathology, Institute of Aquaculture, Hellenic Centre for Marine Research in Athens. Two hundred and twenty-five fish, initial average weight of 35.24 ± 1.47 g, were distributed in groups of 25 fish in nine tanks of 120 l, in triplicate per treatment and left to acclimate for 2 weeks. During the experiment, the photoperiod was 12L:12D light/dark, seawater salinity was 38‰, and temperature was 20 ± 2$^{o}$C. Fish were fed the experimental diets *ad libitum* by hand three times per day (09:00, 12:00, and 15:00), 7 days/week. Two isoenergetic diets were formulated. Diet A, an organically produced feed[37] including sustainable certified fish meal, fish oil, and organic wheat; and Diet B, a laboratory diet including wheat meal and soya meal. A commercial diet, Diet C was also used. The proximate composition of the diets is shown in Table 1. All diets were offered daily to the farmed sea breams *ad libitum* by hand for 41 days.

Wild sea breams (average weight 300 g) were caught in a coastal lagoon of the Aegean Sea, in the area of Kavala and in coastal waters near Pagasitikos Bay. Time of harvest was the same for both cultured and wild fish. At the end of the experiment, fish were weighed and sacrificed by immersion in ice cold water (hypothermia). Their body cavity was opened through a midventral incision and tissue liver samples were immediately obtained from three fish of each dietary treatment and the wild fish. The liver samples were divided equally, the middle part was taken for measurement, and immediately fixed in 2.5% glutaraldehyde in 0.1 M sodium cacodylate solution until further analysis. The hepatosomatic index (HIS) = [liver weight (g)/total weight (g)]x% was calculated for 12 individuals per dietary treatment. Specific growth rates (SGR, %/day), SGR = 100x[ln$W_F$-ln$W_I$/days], where $W_F$ is the final fish weight and $W_I$ is the initial fish weight, were also calculated for the experimental period.

### Preparation of Specimens for Electron Microscopy

Specimens were washed in the same buffer before and after fixation. Postfixation took place using 1% osmium tetroxide in 0.1 M sodium cacodylate for 2 h at 4°C. Specimens were washed in the same buffer before and after postfixation and rinsed in distilled water. Dehydration in a graded alcohol series took place and after that, the specimens were immersed and left overnight in a 1:1 mixture of propylene oxide and the resin used for embedding. The final step of the embedding took place in capsules with agar resin. Polymerization of the resin was completed in 48 h at 60°C.

Ultrathin sections were positively stained. The staining was made with the use of 2% aqueous solution of phosphotungstic acid (PTA; pH 3.3) for 1 h and then with 2% aqueous solution of uranyl acetate (UA; pH 4.3) for 30 min. Before and after UA staining, the ultrathin sections were washed briefly in distilled water.

**TABLE 1**
**Experimental Diet Formulation and Composition[31]**

| Ingredients (g/100 g) | Diet A (Organic) | Diet B (Laboratory) | Diet C (Commercial) |
|---|---|---|---|
| Fishmeal | 63.0 | 55 | 50.0 |
| Fish oil | 9.0 | 9 | 14.0 |
| Wheat meal | — | 25.2 | 11.7 |
| Organic certified wheat meal | 26.5 | | — |
| Soya meal | — | 10 | 16.0 |
| Corn gluten meal | — | | 7.5 |
| Choline | — | | 0.3 |
| Vitamins (premix) | 0.3 | 0.5 | 0.5 |
| *Proximate analysis* | | | |
| Moisture (%) | 10 | 8 | 9 |
| Protein (%) | 45 | 45 | 46 |
| Lipids (%) | 14 | 14 | 17 |
| Fiber (%) | 0.6 | 0.95 | 1.73 |
| Ash (%) | 11 | 6.8 | 9.83 |
| Vitamin A (IU/kg) | 16,000 | | 12,000 |
| Vitamin D (IU/kg) | 1600 | | 1600 |
| Vitamin C (mg/kg) | 715 | | 250 |
| Vitamin E (mg/kg) | 830 | | 825 |
| Zinc oxide (mg/kg) | 107 | | 80 |
| Iron sulfate (mg/kg) | 267 | | 50 |
| Manganese oxide (mg/kg) | 110 | | 70 |
| Cobalt sulfate (mg/kg) | 8 | | 2 |
| Copper sulfate (mg/kg) | 9 | | 4 |
| Inositol | 300 | | 300 |
| P (%) | 1.5 | | — |

## Electron Microscopy

Electron microscopy was performed on a Philips CM-10 electron microscope and micrographs were taken at 13,500X. In order to measure the collagen fibril diameter, the magnification was increased digitally at 43,000X. Grating replicas were used for magnification calibration.

## Measurements of Fibers

The electron micrographs were transferred by a Hewlett Packard scanjet 5530 scanner to a computer in greyscale mode. This transfer was made with a resolution of at least 1200 dots per inch (dpi). Each micrograph was divided into part-images with 500 × 500 pixels dimension. Binary images were produced from the greyscale mode input part-images by thresholding. Collagen fibrils were represented as black against a white background. The cross-sections of some fibrils might not be perpendicular to the fibril axis. Hence, a flat, dish-shaped structuring element with a radius that can be chosen by will were created and applied to the image. The structuring elements were constructed using techniques known collectively as structuring element decomposition. The binary images were processed by watershed morphological algorithm, which allows the identification and labeling of distinct black regions. A vertical and horizontal scanning of the final images was performed in order to measure the dimensions and structural characteristics of each region. Fibril diameter was measured in pixels, which were subsequently converted to nanometers using the dpi of the transferred image. The fibril diameters were measured with the use of an algorithm that was custom built at the Laboratory of Medical Physics at the medical school of Ioannina University[38,39].

### Statistical Analysis

All values are presented as means ± standard deviation and differences present at 5% level are considered significant. The weight means are compared with single factor analysis (ANOVA). When statistically significant differences were confirmed among dietary treatments ($p < 0.05$), further statistical analysis was performed with Tukey' test. Collagen fiber values were checked for normality using the Kolmogorov-Smirnov test. Statistical comparisons between the groups were made using the Kruskal-Wallis one-way analysis of variance (ANOVA) test. Where the above test indicates a significant difference, a Kruskal-Wallis all pairwise comparison test was used to identify which means were significantly different from one another[40].

## RESULTS

The HSI for the sea breams fed the organic diet was 0.96 ± 0.31, the laboratory diet 0.96 ± 0.18, and the commercial diet 0.90 ± 0.35; for the wild sea breams, it was 1.50 ± 0.29. There were no significant differences in the HSI and SGR %/day ($p < 0.05$) between the dietary treatments. The SGR (%/day) for the sea breams fed the organic diet was 1.50 ± 0.16, the laboratory diet 1.54 ± 0.02, and the commercial diet 1.46 ± 0.20. The collagen fibril diameter data distributions from the livers of the wild coastal lagoon fish and from the livers of the fish that were fed the organic diet at the 0.05 level are found to not be drawn from a normal distribution (Kolmonorow Smirnoff test, $p = 0.0051$ and $3.8 \times 10^{-6}$, respectively) (Fig. 1). Furthermore, the Kolmonorow Smirnoff test shows that all the other distributions of collagen fibril diameter data from the livers of the wild coastal fish and from the fish fed with the laboratory and commercial diets at the 0.05 level are drawn from a normal distribution. A nonparametric Kruskal-Wallis ANOVA is also used in order to test if the difference of the means of liver collagen fibril diameter between the wild fish (coastal and lagoon) and from those fed the three diets is statistically important. A $p = 9.1 \times 10^{-92}$ shows that there is a significant difference between treatments.

Liver collagen fibril diameters are given in Table 2. The highest fibril diameters are determined in fish fed the organic diet. Wild fish (coastal) and fish fed the organic diet had similar liver collagen fibril diameters and are not statistically significant ($p > 0.05$) (Table 2, Figs. 2 and 3). Wild fish (lagoon) and fish fed the commercial diet do not appear to have significant differences in their collagen liver fibers ($p > 0.05$) (Table 2, Figs. 2 and 3). However, the collagen fibril diameter data distributions from the livers of the fish fed the laboratory diet are statistically and significantly different than the collagen fiber diameter data distributions from the livers of the fish fed the organic diet ($p < 0.05$) (Table 2).

## DISCUSSION

One of the most important components of connective tissues is collagen. It has a great tensile strength and it is one of the main components of the extracellular matrix of the liver. Along with soft keratin, it is responsible for the tissue's strength and elasticity, and plays a role in tissue development. Transmission electron microscopy and digital image analysis are used widely to study collagen[9,10,11,12,38, 41,42,43,44,45,46,47]. The collagen fibril diameter is regarded as the most important factor related to biomechanical strength of tissues[9,11,46,48,49,50].

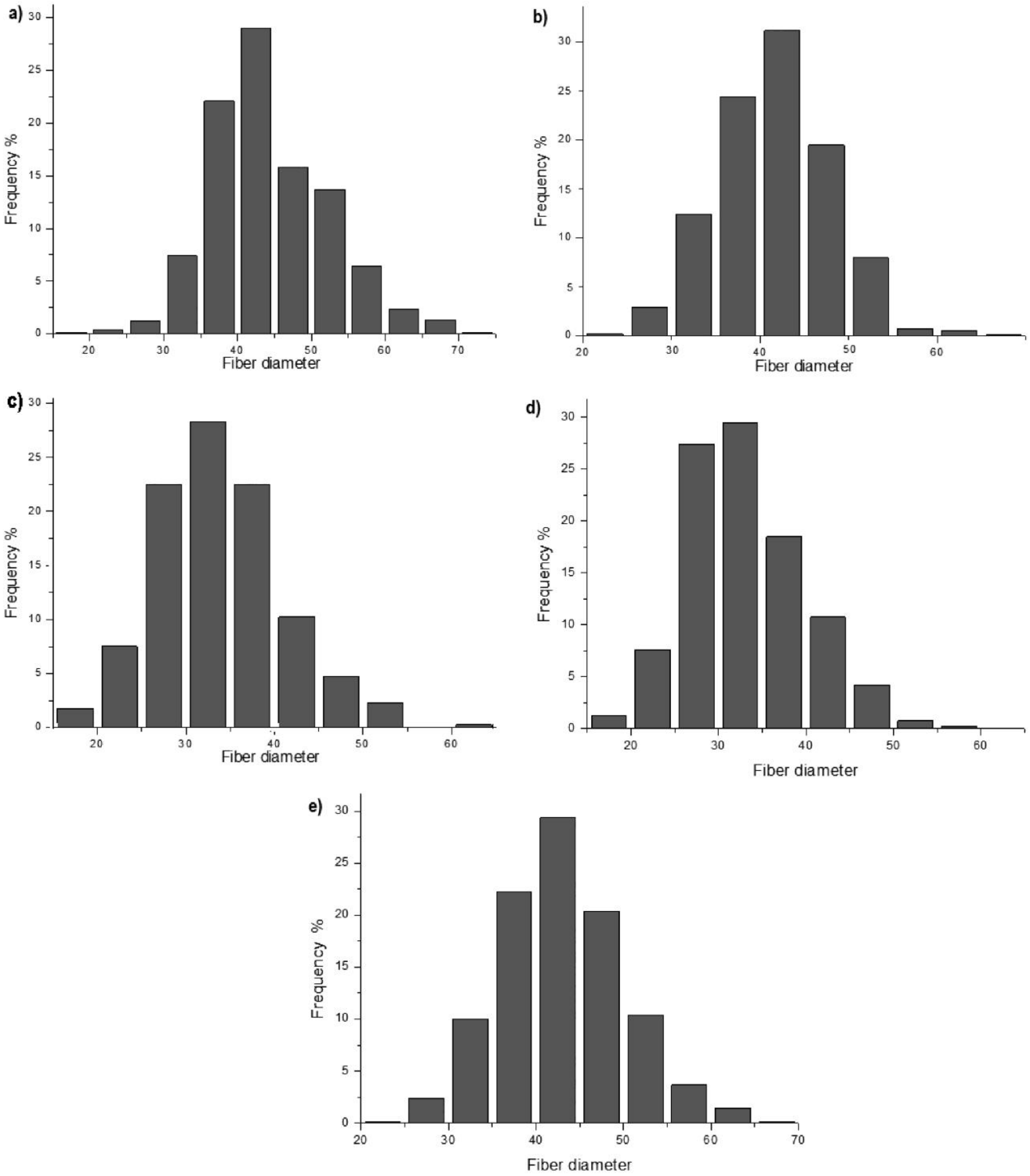

**FIGURE 1.** The distribution of liver collagen fiber size from (a) organic samples, (b) laboratory samples, (c) commercial samples, (d) coastal lagoon samples, and (e) coastal samples in the cultured and wild sea bream (*S. aurata*).

**TABLE 2**
**Mean Diameter of Sea Bream (*S. aurata)* Liver Collagen Fibrils Fed with Different Diets**

| Diets | Collagen Fibril Mean Diameters (nm) |
|---|---|
| Diet A (organic) | 44.27[a] ± 8.03 (n = 729) |
| Diet B (laboratory) | 41.50[b] ± 6.38 (n = 915) |
| Diet C (commercial) | 33.69[c] ± 7.27 (n = 400) |
| Wild sea bream (coastal lagoon) | 32.92[c] ± 6.67 (n = 1044) |
| Wild sea bream (coastal) | 42.87[a,b] ± 7.11 (n = 712) |

Data are presented as means ± S.D.

Means in a column followed by the same superscript are not significantly different ($p > 0.05$).

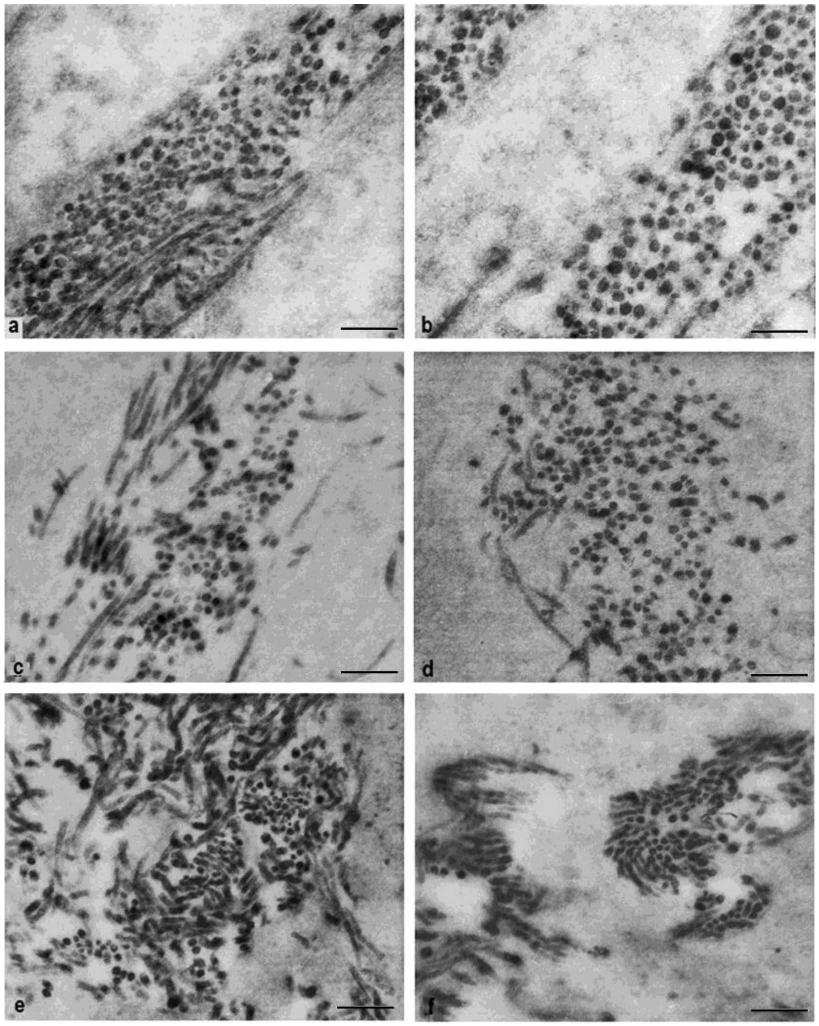

**FIGURE 2.** (a,b) Collagen fibrils from *S. aurata* fed with diet A (organic); (c,d) collagen fibrils from *S. aurata* fed with diet B (laboratory); (e,f) collagen fibrils from *S. aurata* fed with diet C (commercial). Scale bar = 0.23 μm.

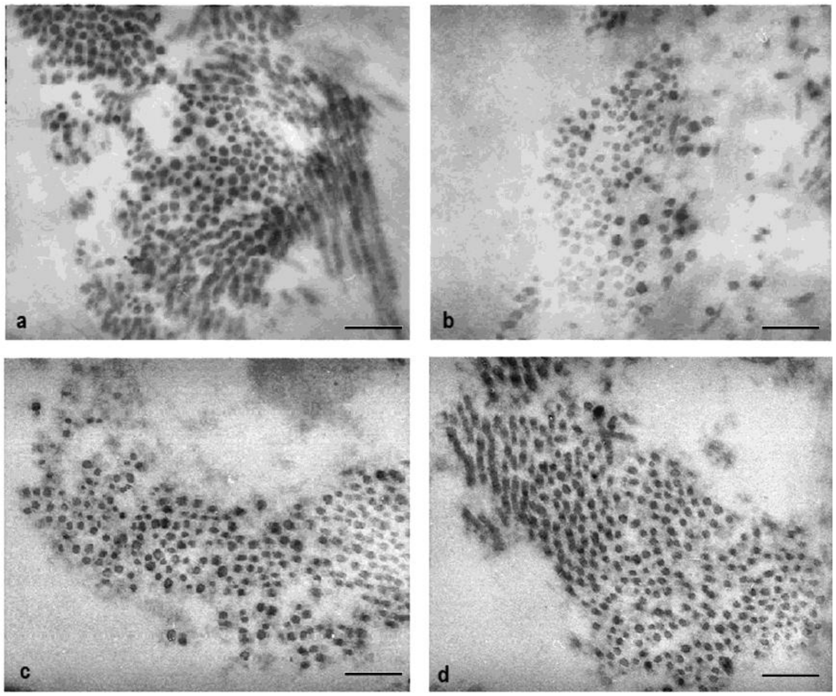

**FIGURE 3.** (a,b) Collagen fibrils from wild (coastal) *S. aurata;* (c,d) collagen fibrils from wild (coastal lagoon) *S. aurata*. Scale bar = 0.23 μm.

Almost one-third of the protein in the body consists of collagen. Collagen plays a key role in providing the structural scaffolding for cells, tissues, and organs[51,52,53,54]. This study shows that the organic diet has higher amounts of vitamins A, C, and E (Table 1). Vitamin C enhances the biosynthesis of collagen[33]. Vitamin A modifies collagen metabolism in different cell types in different ways in rats. In the hepatocyte cells, type I collagen increased with vitamin A[26]. Bone collagen synthesis is significantly inhibited after 24 h of culture with an excess of vitamin A (retinol) and it is progressively decreased, compared with control cultures containing no retinol, as the period of culture is increased[29]. In contrast, fish fed the commercial diet have similar sizes of collagen fibril diameters as the wild fish caught in the coastal lagoon. The coastal lagoon is a semi-intensive production system, where higher yields are sustained by combining the natural food productivity and external feed inputs to supplement dietary nutrients. As supplementary feed, a commercial pelleted feed for sea breams reared in the coastal lagoon is used in this study and confirms the results of the present study.

The composition of commercial feed used for cultured fish also influences the mineral composition of the fish. Fish meal has a relatively high content of minerals, such as phosphorus, and a wide trace of elements, including selenium. Wide variations are observed in the reported values of mineral concentrations in the same species of fish influenced by seasonal and biological differences and food source and environment[55]. Alasalvar et al.[56] show that there is a difference in total lipid content, fatty acid proportions, and trace mineral compositions between white muscle tissues in cultured and wild sea bass *Dicentrachus labrax*. These differences may be attributed to the constituents of the diet of the fish. The above study shows that iron and zinc were predominant elements among 14 minerals analyzed, and constituted 78.2 and 81.6% of the total mineral contents in the flesh of cultured and wild sea bass, respectively.

Vitamin A, zinc, manganese, and selenium aid in collagen formation and metabolism. Selenium- and manganese-deficient diets seem to be responsible for collagen fibrils of decreased diameter[30,34]. Studies show that zinc deficiency decreases collagen turnover and synthesis[28]. Diet supplementation with vitamin A may promote a significant increase in collagen production as a result, especially of type I[26,32]. The manganese diet concentration for the wild sea bream is very important[34]. The organic diet used in this study contained higher amounts of vitamins A and C, zinc oxide, and manganese oxide than did the commercial diet (Table 1). This study confirms that fish fed the organic feed, which had a higher content of vitamins (A, C, and E) and metals (zinc, manganese, iron), resulted in a bigger collagen fibril diameter than fish fed the conventional feed. This study also compared collagen diameters in the livers of wild sea breams with collagen diameters in the livers of farmed fish fed different diets and found that larger collagen liver fibrils are correlated with fish fed diet A and wild fish. Thus, diet A correlates with the natural diet in the wild, and complies with the organic standards in order to maximize fish health and welfare. This work is part of a more extensive study in which the effects of the organic diet and organic aquaculture in growth performance, protein synthesis, amino acid profiles, lipid contents, and microbiological analysis has been monitored. Mente et al.[37] show that fish fed diet A (organic diet) have a good performance, lower lipid content in their white muscle and liver, and an amino acid composition in the white muscle that correlates with the white muscle amino acids in the wild fish, which determines its dietary requirements. The above study aims to develop a better understanding of organic sea bream aquaculture production. It is shown that feed and stocking density have an effect on the final quality product and fish welfare in organic aquaculture.

It is reported that the amino acid composition and amino acid sequence of fish type I collagens are quite different from those of higher vertebrates[36,57,58]. Therefore, it is likely that the functionality of fish collagen as a food material is different from that of higher vertebrate collagen. Fish collagen peptide contains large amounts of glycine, proline, hydroxyproline, glutamic acid, alanine, lysine, and arginine (Table 3). In the present study, the organic feed contains more glycine, alanine, and lysine than the conventional feed. Kaushik[59] calculates the theoretical essential amino acid requirement profile of gilthead sea bream and sea bass, and finds that the requirements for both species were higher for lysine, arginine, and leucine. Using body composition of wild sea bream as the ideal amino acid profile, the lysine requirements are higher than the other essential amino acids (Table 3). The amino acid profile of the conventional diet (diet C), particularly for the lysine, alanine, and glycine content, appears to be unsatisfactory (Table 3). Saito et al.[36] reported that plasma triglyceride reduction in rats owing to fish collagen peptide intake has a close relationship with the concentration of amino acid–derived fish collagen peptides. Thus, there is an effect on lipid metabolism by the amino acids and/or peptides derived from consumption of fish collagen peptide. Fish collagen peptides are widely used as a protein additive in nutraceutical food and cosmetics. Collagen peptides derived from fish skin, scales, and bone, and the processing of fish wastes from several species, can be used as protein additives in aquafeeds and in organic aquaculture. As a general principle in nutrition of organically farmed aquatic animals, diets must meet the nutritional requirements of the organisms, promote the animal’s growth and health, ensure high quality of the final edible product, and cause low environmental impact[60]. The development of nutritionally efficient diets using organic sources of ingredients in organic aquaculture diets is a challenge.

Aquaculture production has raised concerns in recent years over the quality of cultured fish in comparison with that of wild fish. Comparisons between cultured and wild fish in terms of their fatty acid compositions confirm that their fatty acid compositions are different[20,61]. Previous findings suggest that sea bream produced in aquaculture have lower moisture content and higher lipid content compared to wild fish, and protein content is not significantly different between the farmed and the wild fish[55,62]. Differences observed depend on their reduced activity and their diet consumed since there is a high dietary fat level in the feed. Fat deposition in fish tissues is affected by many factors, including dietary lipid content, which has been positively related to the fat content in tissues[61]. Mente et al.[37] show that organic cultured sea breams store less fat content in their white muscle and they grow better compared with the conventional sea breams (liver lipid content is lower, while HSI is higher for the organic sea breams). Moreover, neither moisture nor protein contents of the organic fish differed significantly from the wild fish, unlike the conventional fish. Hence, the lower dietary lipid content of the organic diet has probably resulted in the lower lipid content of the organic sea bream white muscle and liver in comparison

**TABLE 3**
**Amino Acid Profiles of Organic and Conventional Diet**

| Amino Acids | | Organic Diet[1] (% of total) | Conventional Diet[1] (% of total) | Amino Acids[2] (g/16 g N diet) | Collagen Peptides[3] (Residues/1000) |
|---|---|---|---|---|---|
| Aspartic acid | Asp | 7.9 | 8.1 | | 53 |
| Glutamic acid | Glu | 11.89 | 12.74 | | 79 |
| Hydroxyproline | Hyp | — | — | | 55 |
| Serine | Ser | 5.02 | 5.47 | | 51 |
| Glycine | Gly | 10.03 | 8.63 | | 369 |
| Histidine | His | 1.63 | 1.44 | 1.70 | 8 |
| Arginine | Arg | 3.99 | 3.9 | 5.40 | 65 |
| Threonine | Thr | 5.2 | 4.69 | 2.80 | 20 |
| Alanine | Ala | 10.36 | 9.62 | | 100 |
| Proline | Pro | 5.81 | 6.86 | | 104 |
| Tyrosine | Tyr | 2.37 | 2.59 | | |
| Valine | Val | 6.55 | 6.17 | | 14 |
| Methionine | Met | 2.83 | 2.26 | 2.40 Met+Cys | 12 |
| Isoleucine | Ile | 5.06 | 5.06 | 2.60 | 10 |
| Leucine | Leu | 8.87 | 10.52 | 4.50 | 16 |
| Phenylalanine | Phe | 4.13 | 4.89 | 2.90 Phe+Tyr | 12 |
| Lysine | Lys | 8.36 | 7.07 | 5.00 | 22 |

[1] Amino acid composition of cultured sea bream[24].

[2] Data on available dietary amino acid needs in gilthead sea bream[56].

[3] Salmon collagen peptides[22].

to the conventional group. In this study, fish fed the organic feed are shown to have significantly bigger collagen fibril diameters than fish fed the conventional feed. Furthermore, the organically fed fish have similar size collagen fibril diameters as the wild coastal fish.

To our knowledge, this is the first demonstration that collagen fibril diameters measured in the liver are affected by the nutritional status of the fish. The liver is an organ that can be used to evaluate if variations in dietary profiles may affect fish health and condition[63]. The liver is a target organ in the study of diet-induced effects as it is the main metabolic organ and plays an important role in fish immune responses. Diet can affect fish liver collagen fibril diameters since it contains some trace elements and vitamins that play an important role in collagen synthesis, metabolism, and fibril diameter. More research is needed in order to understand the long-term effect and the mechanism and function of fish collagen peptide intake on lipid absorption and metabolism, and then to identify dietary regimes that will be able to improve whole body lipid profiles and suppress transient increase of plasma triglycerides.

## ACKNOWLEDGMENTS

This research was cofunded by the European Commission and the Hellenic Ministry of Rural Development and Food, Fisheries and Aquaculture Department (EPAL). The authors wish to express their thanks to G. Varvatsoulis, Bitsakos Aquaculture Farm, and Zoonomi Aquafeed Company for their help.
